# Heat hardening enhances mitochondrial potential for respiration and oxidative defence capacity in the mantle of thermally stressed *Mytilus galloprovincialis*

**DOI:** 10.1038/s41598-021-96617-9

**Published:** 2021-08-24

**Authors:** Ioannis Georgoulis, Konstantinos Feidantsis, Ioannis A. Giantsis, Asimina Kakale, Christian Bock, Hans O. Pörtner, Inna M. Sokolova, Basile Michaelidis

**Affiliations:** 1 Laboratory of Animal Physiology, Department of Zoology, School of Biology, Aristotle University of Thessaloniki, 54124 Thessaloniki, Greece; 2 Department of Animal Science, Faculty of Agricultural Sciences, University of Western Macedonia, 53100 Florina, Greece; 3 Alfred-Wegener-Institut, Helmholtz-Center for Polar and Marine Research, Integrative Ecophysiology, Postfach 120161, 27515 Bremerhaven, Germany; 4 Department of Marine Biology, Institute for Biological Sciences, University of Rostock, A.-Einstein Str., 3, 18055 Rostock, Germany

**Keywords:** Animal physiology, Climate change

## Abstract

Ectotherms are exposed to a range of environmental temperatures and may face extremes beyond their upper thermal limits. Such temperature extremes can stimulate aerobic metabolism toward its maximum, a decline in aerobic substrate oxidation, and a parallel increase of anaerobic metabolism, combined with ROS generation and oxidative stress. Under these stressful conditions, marine organisms recruit several defensive strategies for their maintenance and survival. However, thermal tolerance of ectothermic organisms may be increased after a brief exposure to sub-lethal temperatures, a process known as "hardening". In our study, we examined the ability of *M. galloprovincialis* to increase its thermal tolerance under the effect of elevated temperatures (24, 26 and 28 °C) through the "hardening" process. Our results demonstrate that this process can increase the heat tolerance and antioxidant defense of heat hardened mussels through more efficient ETS activity when exposed to temperatures beyond 24 °C, compared to non-hardened individuals. Enhanced cell protection is reflected in better adaptive strategies of heat hardened mussels, and thus decreased mortality. Although hardening seems a promising process for the maintenance of aquacultured populations under increased seasonal temperatures, further investigation of the molecular and cellular mechanisms regulating mussels’ heat resistance is required.

## Introduction

Climate change, leading to an increase in sea surface temperatures, affects marine organisms at all levels of biological organization, including molecular, biochemical, and physiological^1–3^. Ectotherms are particularly sensitive to these changes due to the direct effect of ambient temperature on the body temperature, and thus on the rates of all biochemical and physiological reactions. According to the OCLTT hypothesis, temperature increase beyond organism’s optimum limits (at pejus temperatures, Tp) leads to a mismatch between energy demand and energy supply from aerobic pathways, a compensatory shift to partial anaerobiosis, and energy deficiency^3–6^. Furthermore, the deviation of temperature from optimum conditions negatively affects the cellular redox balance because supraoptimal (pejus) temperatures lead to elevated electron leak from the ETS in ectotherms’ mitochondria^7,8^, further enhanced by temperature-induced oxygen deficiency (hypoxemia)^3,4,8,9^. Oxidative stress caused by excessive ROS production can lead to cellular damage and eventually cell death, so that mitigation of the oxidative stress via adjustment of mitochondrial ETS activity and/or upregulation of antioxidants plays a key role in the survival and contributes to the costs of cellular homeostasis under heat stress^8,10–14^.

Thermal tolerance of organisms is a plastic trait and can be modified by acclimation to different thermal regimes, as well as by brief exposures to sub-lethal temperatures which are known as heat hardening^15^. Heat hardening is defined as a transient response that confers improved heat tolerance immediately after the initial heat-stress bout for up to 32 h, while longer-lasting improvements in heat tolerance are termed as heat acclimation^15,16^. This adaptive response was firstly described by Precht^17^, who defined “*hardening*” as a rapid beneficial response occurring within a few hours in contrast to acclimation that may take days. Heat hardening might be considered as a special case of a broader phenomenon of stress hormesis (also termed stress priming, preconditioning or acquired stress response), where a mild stress confers enhanced resistance toward higher levels of the same stress or to a stressor of a different nature^18^. The mechanisms of heat hardening are not yet fully understood but involve co-activation of multiple stress signaling pathways (including reactive oxygen, nitrogen and carbonyl species, unfolded protein response and transcription factors) that lead to phenotypes with increased resistance^19–22^. Mitochondria play a key role in hormetic responses such as heat hardening by releasing ROS and other signaling molecules that induce the cellular stress response^8,23^. Activation of stress signaling pathways leads to a concerted cellular response at transcriptional and post-transcriptional levels that restore metabolic, proteome, and redox homeostasis, and can protect the organism against subsequent stress impacts^24–27^.

**Figure 1:**
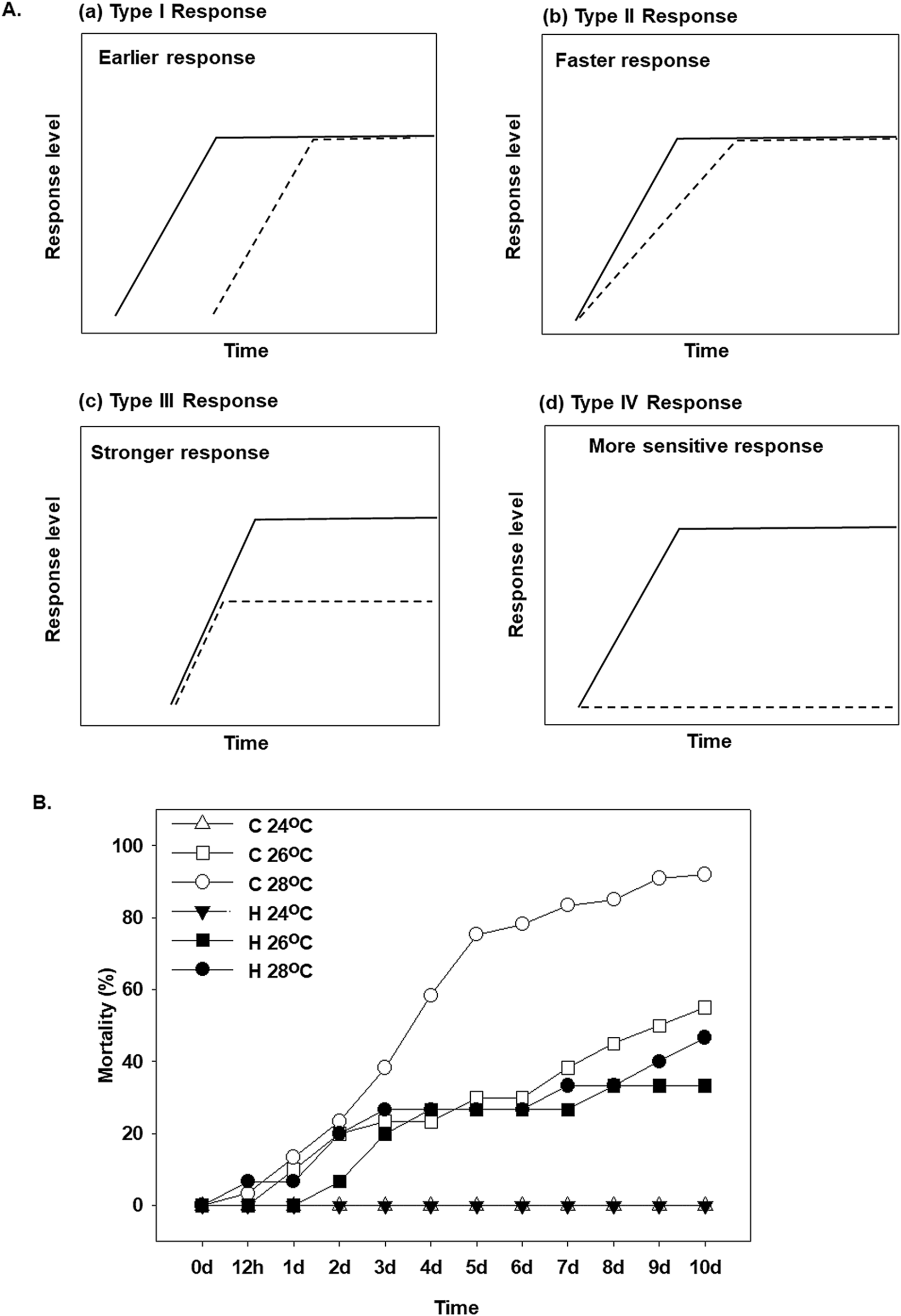
(**A**) Four main types of stress responses depending on the amplitude and the time course, involved in hardening have been described in Conrath et al. ^28^ and Hilker et al. ^18^: (a) an earlier stress response (type I response), (b) a faster stress response (type II response), (c) a stronger stress response (type III response) and (d) a higher sensitivity hardening stress response (type IV response). Continuous lines indicate primed response to stress while dashed lines indicate non-primed response to stress. (**B**) Effect of water temperature (24, 26 and 28 °C) on the mortality of heat hardened (H) and non-hardened (C) *Mytilus galloprovincialis* compared to control at 18 °C.

Depending on the amplitude and the time course, four main types of stress responses involved in hardening have been described^18,28^ (Fig. 1A). An earlier-onset (type I response) hardened stress response exhibits the same kinetics of stress pathway activation as in a naïve organism exposed to stress, but with a shorter lag phase until the stress response starts to build up in a preconditioned organism. Consequently, the final defense level is reached earlier in hardened than in the naïve state. In a faster hardening stress response (type II response), an hyperactivation and a faster signaling cascade is observed, leading to a more rapid buildup of the stress defense in a preconditioned organism. The stronger stress response (type III response) in a hardened organism initially resembles the naïve response, but activation of stress response mechanisms reaches a higher final level than in the naïve organism. Under these conditions, hyperactivation and an enhanced gene expression could be responsible, resulting in a higher response amplitude. Higher sensitivity hardening stress response (type IV response), indicating a triggering response in lower stress intensity in contrast with naïve organism. These changes in the expression pattern and thresholds of the transcriptional induction of stress response genes during heat hardening play an important role in development of the heat-tolerant phenotypes of animals^29–33^. Such phenotypic plasticity in response to heat hardening have been proposed as an adaptive strategy under the extreme heat events predicted with climate change for marine mussels including *Mytilus californianus* (Conrad, 1837)^34^. However, the physiological and molecular mechanisms of heat hardening are not well understood in marine ectotherms, including mussels, and require further investigation to assess the potential mechanisms underlying the organism’ ability to cope with extreme weather events such as marine heat waves.

The Mediterranean mussel *M. galloprovincialis* (Lamarck, 1819) is an ecologically and economically important species of marine bivalves and is used in research on stress responses to warming. Previous studies showed that *M. galloprovincialis* is thermally stressed when exposed to 26 °C–27 °C which results in gradually increased mortality^35,36^. The aim of the present study was to investigate whether heat hardening enhances thermal tolerance of *M. galloprovincialis,* and whether transcriptional and/or post-transcriptional regulation of the pathways involved in mitochondrial energy metabolism, antioxidant defense, and protein quality control are implicated in this phenomenon. To assess the impact of acute heat stress and heat hardening on mitochondrial bioenergetics, we examined mRNA expression of mitochondrial encoded subunits of complex I and IV of ETS (*ND2* and *COX1*, respectively) and determined the ETS activity in the mantle of control, heat stressed and heat-hardened mussels. ETS activity is an indicator of the maximal velocity (*V*_max_) of the ETS multi-enzyme complexes and is considered a biochemical measure of the metabolic potential of organisms^13^. To assess the cellular energy status, levels of phosphorylated AMPK (a key energy sensor activated in response to low cellular ATP levels) were measured. To determine the potential involvement of oxidative stress and redox signaling in heat hardening, we determined mRNA expression of enzymatic *CuSOD*, *MnSOD*, *GST*, *CAT,* and non-enzymatic (*mt10*) antioxidants, and measured activities of the antioxidant enzymes SOD, GR, and CAT. Furthermore, mRNA and protein expression of a key molecular chaperone (Hsp70) involved in refolding of heat-damaged proteins was assessed to determine the potential relationship between antioxidant defense and protein quality control mechanisms during heat hardening in *M. galloprovincialis*.

## Results

### Mortality

At 24 °C, both hardened and non-hardened mussels exhibited no mortality (Fig. 1B). Temperature increase to 26 °C elevated mortality to 25–30% in the first 6 days of exposure. While non-hardened mussels exhibited 55% mortality after 10 days of exposure to 26 °C, the cumulative mortality of hardened mussels was lower, reaching 40% by day 15. At the highest tested temperature (28 °C), mortality of non-hardened mussels was 85% in the first week and 100% by day 10. The mortality of hardened mussels at 28 °C was 30% in the first 7 days and reached 60% by day 10 (Fig. 1B).

### ETS activity

**Figure 2:**
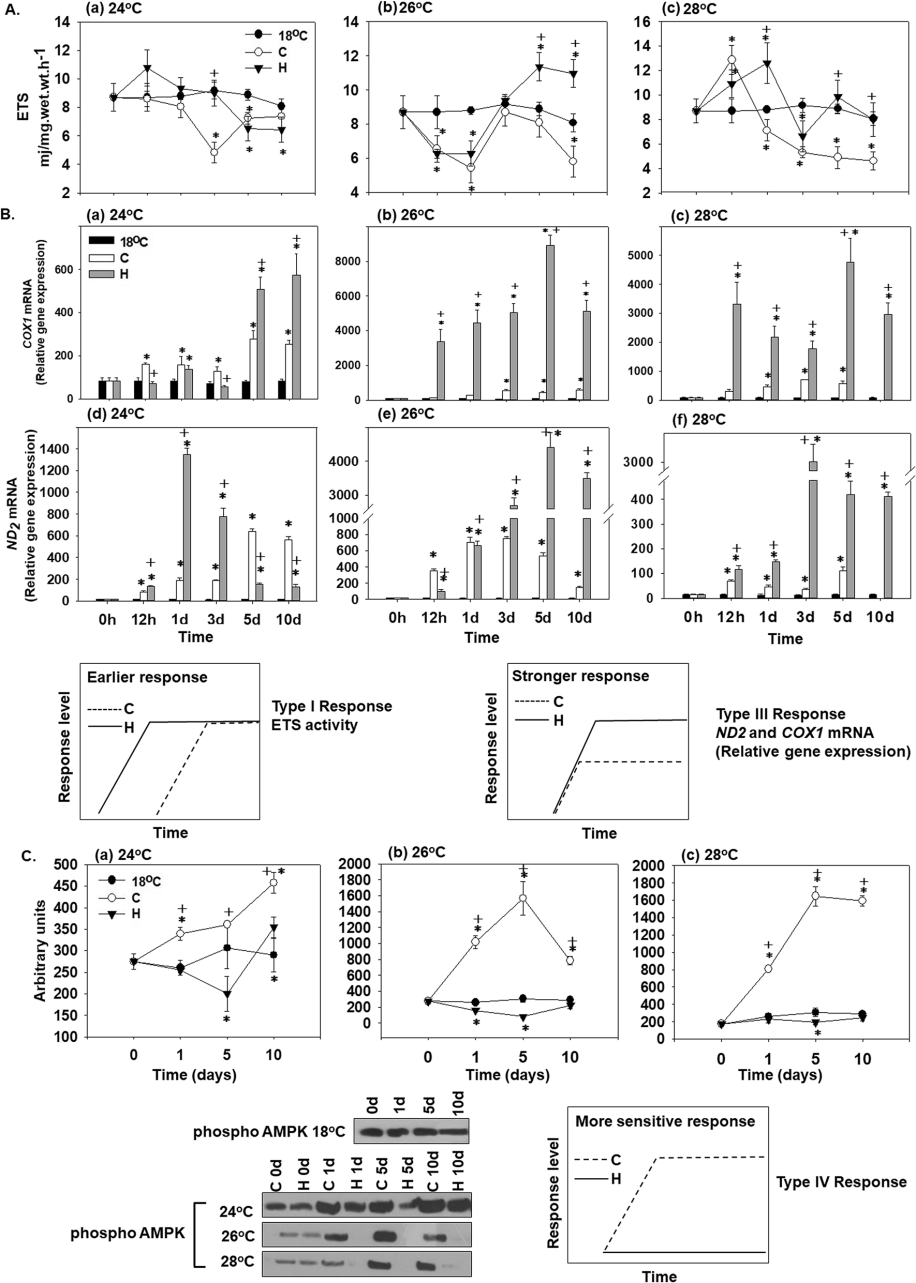
Changes in (**A**) the ETS activity [Type I (earlier) response], (**B**) the relative *COX1* and *ND-2* mRNA levels [Type III (stronger) response] and (**C**) AMPK phosphorylation levels [Type IV (more sensitive) response] in the mantle of heat hardened (H) and non-hardened (C) *Mytilus galloprovincialis* when exposed to 24 °C (a,d), 26 °C (b,e) and 28 °C (c,f) compared to control at 18 °C. Representative blots are shown. Blots were quantified using scanning densitometry. Full-length blots/gels are presented in Supplementary Fig. 1. Values are means ± SD, n = 8 preparations from different animals. *p < 0.05 compared to day 0, ^+^*p* < 0.05 compared to non-hardened (C) mussels.

At 24 °C, there was a time-dependent decrease in ETS activity in both hardened and non-hardened mussels during 10 days of exposure (Fig. 2Aa). ETS activity levels were similar in hardened and non-hardened mussels at 24 °C, and did not significantly differ from the control levels in the mussels kept at 18 °C (Fig. 2Aa). Exposure to 26 °C resulted in a decrease of ETS activity on day 1 compared to the control (Fig. 2Ab). Thereafter, ETS activity levels increased in both experimental groups on day 3 but this increase was transient in the non-hardened mussels followed by a decrease on days 5–10, whereas in the hardened mussels the ETS activity continued to increase reaching significantly higher levels than in the non-hardened and control mussels on days 5 and 10 (Fig. 2Ab). At 28 °C, ETS activity of non-hardened mussels showed a transient increase after 12 h of exposure followed by a continual decrease throughout the exposure, so that after 1–10 days of exposure the ETS activity of non-hardened heat-stressed mussels were significantly below the baseline (control) levels (Fig. 2Ac). The hardened mussels exhibited increased ETS activity after 12 h and 1 day of 28 °C exposure followed by a decrease back to the baseline (control) levels (Fig. 2Ac). As a result, the ETS levels after 1–10 days of exposure at 28 °C were higher in the hardened compared with the non-hardened mussels.

Statistically significant differences were observed between hardened and non-hardened specimens. Main effects of treatment and exposure time, as well as factor interactions, were significant (*p* < 0.0001).

### *COX1* and *ND2* gene expression

Elevated temperatures (24, 26 and 28 °C) caused increased *COX1* and *ND2* mRNA expression in hardened (H) and non-hardened (C) mussels from the 1st day of exposure compared to the baseline levels observed in the control mussels maintained at 18 °C (Fig. 2B). During early exposure (12 h–3 days) to 24 °C, *COX1* mRNA expression was slightly but significantly higher in non-hardened mussels compared to their heat-hardened counterparts (Fig. 2Ba). On days 5 and 10 of exposure to 24 °C, *COX1* mRNA levels were considerably higher in the hardened than in the non-hardened mussels (Fig. 2Ba). The mRNA levels of *ND2* peaked at day 1 of exposure to 24 °C in the hardened mussels reaching significantly higher levels than in their non-hardened counterparts (Fig. 2Bd). Later during exposure to 24 °C, the levels of *ND2* decreased in the hardened mussels whereas they continued to rise in non-hardened mussels so that on days 5 and 10 of exposure to 24 °C the *ND2* transcript levels were higher in the non-hardened than hardened mussel. It is worth noting that in both hardened and non-hardened groups the *COX1* and *ND2* transcript levels were significantly higher than baseline (control) levels at all studied time points. At 26 °C and 28 °C, *COX1* and *ND2* mRNA expression levels were consistently and significantly higher in hardened (H) compared to non-hardened (C) mussels at all studied time points (Fig. 2Bb,c,e,f).

Statistically significant differences were observed between hardened and non-hardened specimens. Main effects of treatment and exposure time, as well as factor interactions, were significant (*p* < 0.0001).

### AMPK phosphorylation

Protein expression levels of phosphorylated AMPK were statistically increased in non-hardened (C) mussels from the 1st day of exposure to elevated temperatures (24, 26 and 28 °C) compared to the baseline levels in the mussels kept at 18 °C. The hardened mussels (H) showed low expression levels of phosphorylated AMPK at elevated temperatures (24, 26 and 28 °C) that were consistently lower than in the non-hardened mussels, and often below the baseline levels found in the mussels kept at 18 °C (Fig. 2C).

Statistically significant differences were observed between hardened and non-hardened specimens. Main effects of treatment and exposure time, as well as factor interactions, were significant (*p* < 0.0001).

### *SOD* mRNA expression and activity

**Figure 3:**
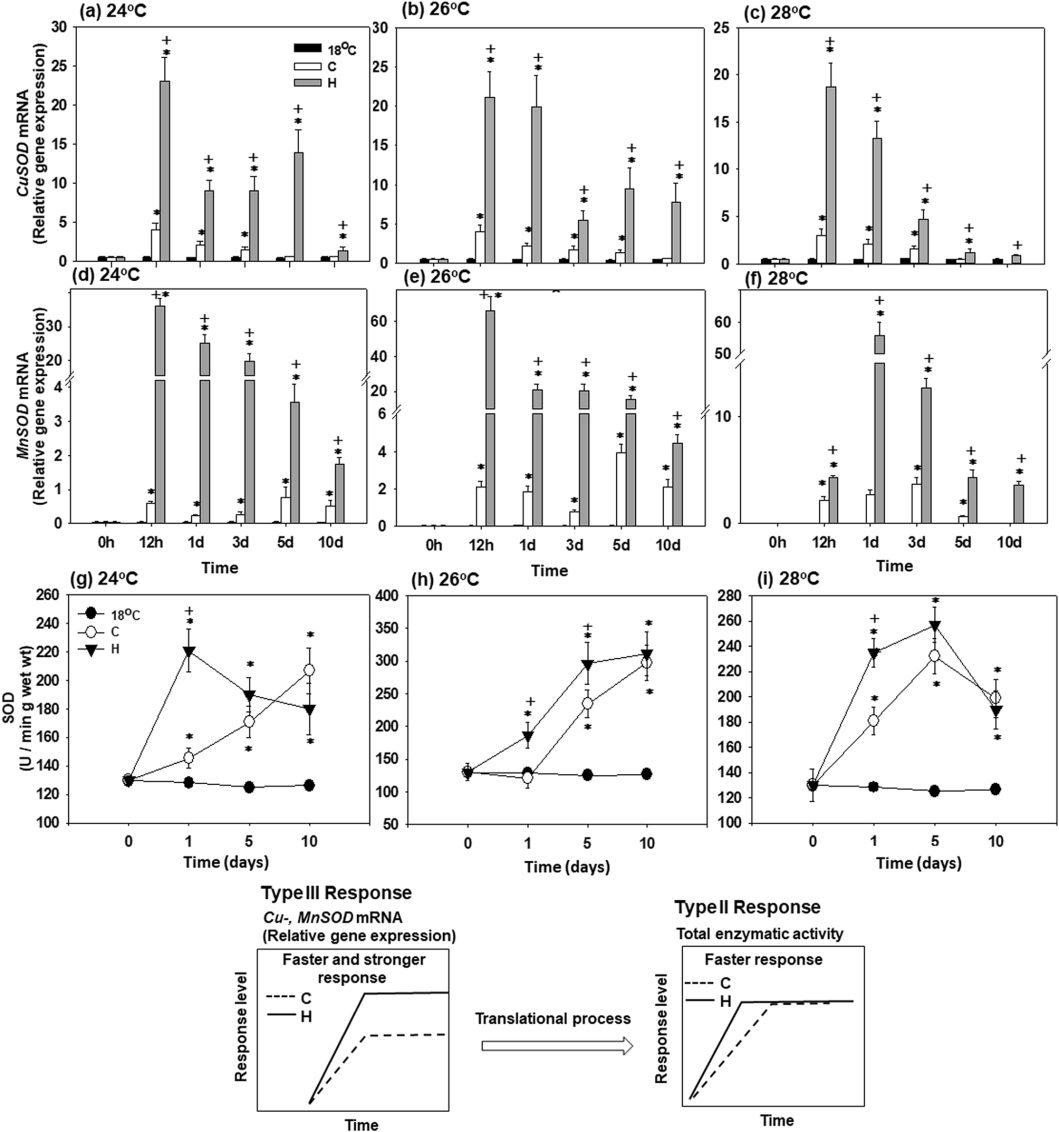
Changes in the relative *CuSOD* and *MnSOD* mRNA levels [Type III (stronger) response], and SOD enzymatic activity [Type II (faster) response] in the mantle of heat hardened (H) and non-hardened (C) *Mytilus galloprovincialis* when exposed to 24 °C (**a**,**d**,**g**), 26 °C (**b**,**e**,**h**) and 28 °C (**c**,**f**,**i**) compared to control at 18 °C. Values are means ± SD, n = 8 preparations from different animals. *p < 0.05 compared to day 0, ^+^*p* < 0.05 compared to non-hardened (C) mussels.

*CuSOD* mRNA expression increased in hardened (H) mussels exposed to all three elevated temperatures (24, 26 and 28 °C) starting from 12 h of exposure (Fig. 3a–c). The expression levels of *CuSOD* mRNA dropped later on (during days 1–10) of heat exposure in the hardened mussels but remained statistically higher compared to non-hardened (C) mussels and those kept at 18 °C. A similar time course with an early (12 h) increase and subsequent decline in *CuSOD* mRNA was observed in the non-hardened (C) mussels albeit the transcript levels remained considerably lower than in the hardened mussels (except for 10 days at 28 °C) (Fig. 3a–c).

*MnSOD* mRNA expression in hardened (H) mussels peaked at 12 h at 24 °C and 26 °C, and at 1 day at 28 °C (Fig. 3d–f). The transcript levels of *MnSOD* decreased later during heat exposure in the hardened mussels (starting on day 1 at 24 °C and 26 °C, and day 3 at 28 °C) but remained statistically higher compared to non-hardened (C) mussels and those kept at 18 °C (Fig. 3d–f). In the non-hardened mussels, *MnSOD* mRNA levels increased later and to a lesser degree than in the hardened counterparts. Thus, at 24 °C and 26 °C, *MnSOD* transcript levels of non-hardened mussels increased significantly above the baseline on days 5 and 10 only (Fig. 3d,e). At 28 °C, the expression levels of *MnSOD* mRNA increased above the baseline already at 12 h of exposure, but the degree of stimulation was considerably lower than in the hardened mussels (Fig. 3f).

SOD enzymatic activity in the mantle of the hardened (H) mussels was elevated above the baseline starting on day 1 and remained elevated at days 5 and 10 of exposure to 24 °C (Fig. 3g). In non-hardened mussels the increase in the SOD enzymatic activity was slower with a mild (but statistically significant) increase above the baseline on day 1 and continual rise throughout the rest of the experimental exposure. As a result, SOD activities of both hardened and non-hardened groups reached similar levels at days 5 and 10 of exposure to 24 °C. At 26 °C and 28 °C (Fig. 3h,i), the dynamics of SOD activity changes were similar in the hardened (H) and non-hardened (C) mussels, except in non-hardened mussels at day 1 to 26 °C. An increase throughout the exposure period (at 26 °C) or an increase after days 1–5 was followed by a decrease at day 10 (at 28 °C). However, the amplitude of the increase in the SOD activity was higher in the hardened (H) compared to the non-hardened (C) mussels at 26 and 28 °C except for day 10 of the respective exposures (Fig. 3h,i).

Statistically significant differences were observed between hardened and non-hardened specimens. Main effects of treatment and exposure time, as well as factor interactions, were significant (*p* < 0.0001).

### *CAT* mRNA expression and activity

**Figure 4:**
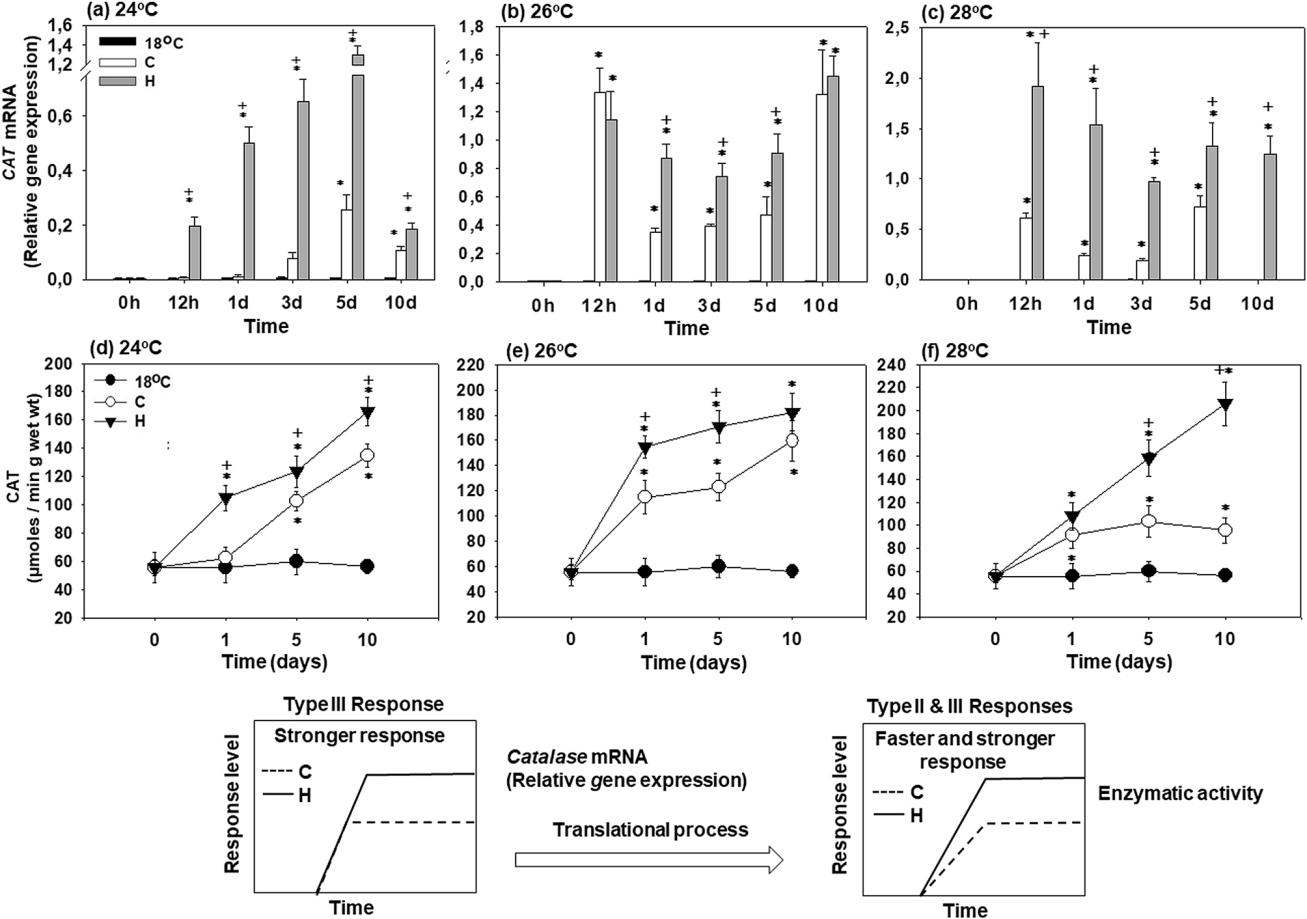
Changes in the relative *CAT* mRNA levels [Type III (stronger) response] and CAT enzymatic activity [Type II & III (faster and stronger) responses] in the mantle of heat hardened (H) and non-hardened (C) *Mytilus galloprovincialis* when exposed to 24 °C (**a**,**d**), 26 °C (**b**,**e**) and 28 °C (**c**,**f**) compared to control at 18 °C. Values are means ± SD, n = 8 preparations from different animals. *p < 0.05 compared to day 0, ^+^*p* < 0.05 compared to non-hardened (C) mussels.

In the hardened (H) mussels, *CAT* mRNA expression increased significantly above the baseline levels after 12 h of exposure at all tested temperatures (24, 26 and 28 °C) and remained strongly elevated until day 10 (Fig. 4a–c). In the non-hardened (C) mussels, transcriptional upregulation of *CAT* occurred later (at 28 °C) and/or to a lesser degree (at all three studied temperatures) than in their hardened counterparts. Transcript levels of *CAT* in heat-exposed hardened and non-hardened mussels were significantly above the baseline levels (measured in the mussels maintained at 18 °C) at all experimental temperatures and time points (Fig. 4a–c). Generally, transcriptional upregulation of *CAT* was higher in the hardened than in the non-hardened mussels except for 12 h, and 10 days at 26 °C where similar levels were attained in these two groups (Fig. 4b).

In parallel with the mRNA expression, CAT enzymatic activity increased at all elevated temperatures (Fig. 4d–f). In the hardened (H) mussels, CAT activity continuously increased throughout the 10 days of exposures at 24, 26 and 28 °C. In the non-hardened (C) mussels, CAT activity increased during 10 days of heat exposure at 24 °C and 26 °C, whereas at 28 °C, a modest increase at days 1–5 of exposure was followed by a decrease at day 10 (Fig. 4f). In both hardened and non-hardened mussels, CAT activity during heat exposure was higher than the baseline measured in the mussels at 18 °C, and CAT activity was higher in the hardened compared to the non-hardened mussels (Fig. 4d–f).

Statistically significant differences were observed between hardened and non-hardened specimens. Main effects of treatment and exposure time, as well as factor interactions, were significant (*p* < 0.0001).

### *GST* mRNA expression

**Figure 5:**
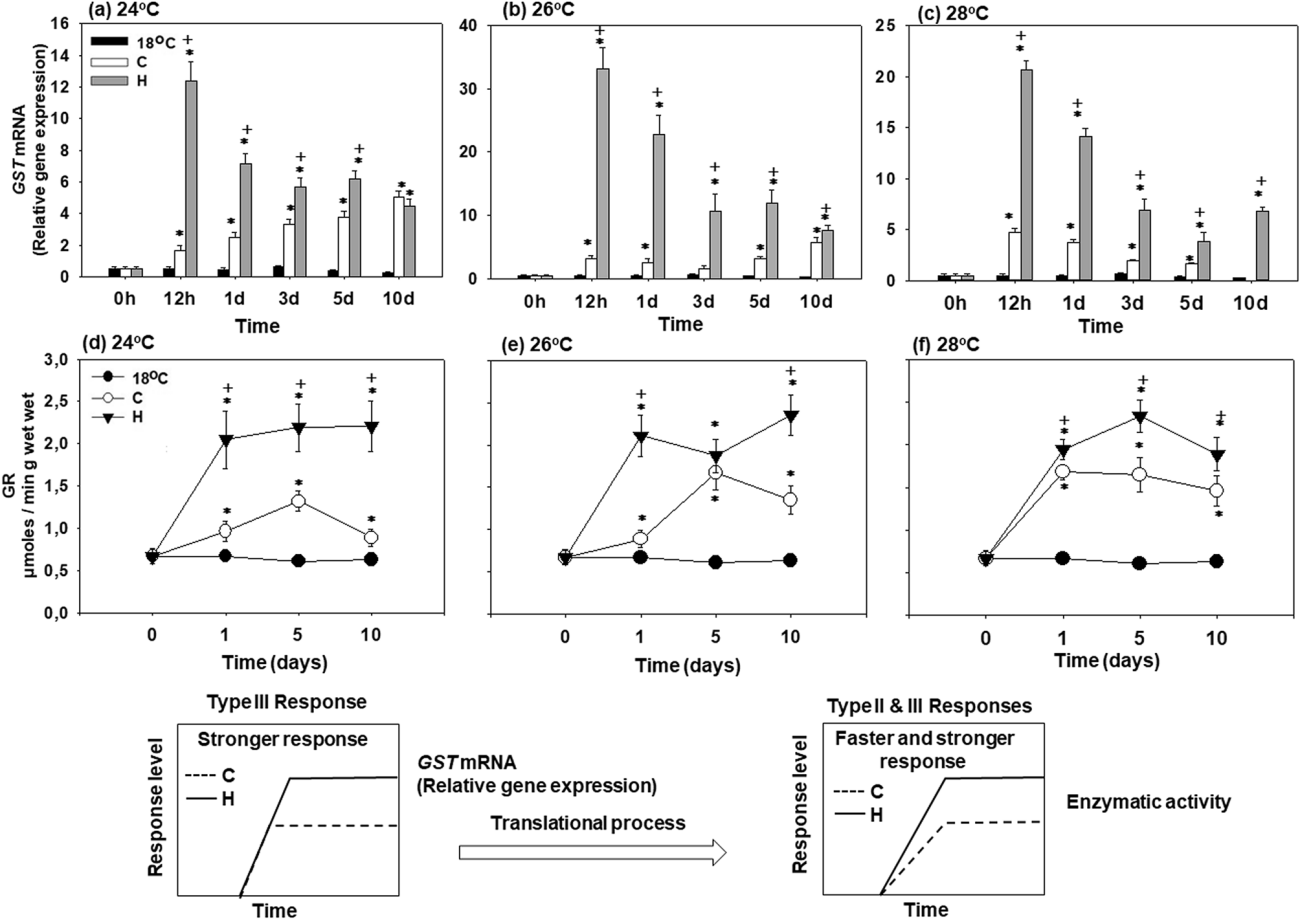
Changes in the relative *GST* mRNA levels [Type III (stronger) response] and GR enzymatic activity [Type II & III (faster and stronger) responses] in the mantle of heat hardened (H) and non-hardened (C) *Mytilus galloprovincialis* when exposed to 24 °C (**a**,**d**), 26 °C (**b**,**e**) and 28 °C (**c**,**f**) compared to control at 18 °C. Values are means ± SD, n = 8 preparations from different animals. *p < 0.05 compared to day 0, ^+^*p* < 0.05 compared to non-hardened (C) mussels.

*GST* mRNA expression of hardened (H) mussels strongly increased after 12 h of exposure to elevated temperatures (24, 26 or 28 °C), and decreased afterwards while remaining significantly above the baseline levels (measured in the non-exposed mussels at 18 °C) at all experimental time points (Fig. 5a–c). In the non-hardened mussels, a significant transcriptional upregulation of *GST* above the baseline levels was observed at day 1 (24 °C), day 5 (26 °C) and 12 h (28 °C) (Fig. 5a–c). At 24 °C and 26 °C, *GST* mRNA expression increased continuously in non-hardened (C) mussels throughout 10 days of experimental exposure (Fig. 5a,b). At 28 °C, *GST* mRNA expression peaked after 12 h and decreased afterwards reaching basal levels after 10 days of exposure (Fig. 5c). At all test temperatures, *GST* mRNA expression in hardened (H) mussels was significantly higher compared to the non-hardened (C) ones throughout the experimental exposure (Fig. 5a–c).

Statistically significant differences were observed between hardened and non-hardened specimens. Main effects of treatment and exposure time, as well as factor interactions, were significant (*p* < 0.0001).

### *GR* enzymatic activity

GR activity increased in hardened (H) and non-hardened (C) mussels during exposure to elevated temperatures compared with baseline levels found in mussels kept at 18 °C (Fig. 5d–f). In the hardened mussels, the GR activity increased on day 1 and remained elevated during 10 days of experimental exposure (Fig. 5d–f). In the non-hardened mussels, an initial increase of GR activity (on day 5 at 24 °C and 26 °C, and on day 1 at 28 °C) was followed by a decrease later during exposures (Fig. 5d–f). At all experimental time points, GR activity in the hardened mussels was higher than in their non-hardened counterparts (Fig. 5d–f).

Statistically significant differences were observed between hardened and non-hardened specimens. Main effects of treatment and exposure time, as well as factor interactions, were significant (*p* < 0.0001).

### *Metallothionein-10 (mt-10)* gene expression

**Figure 6:**
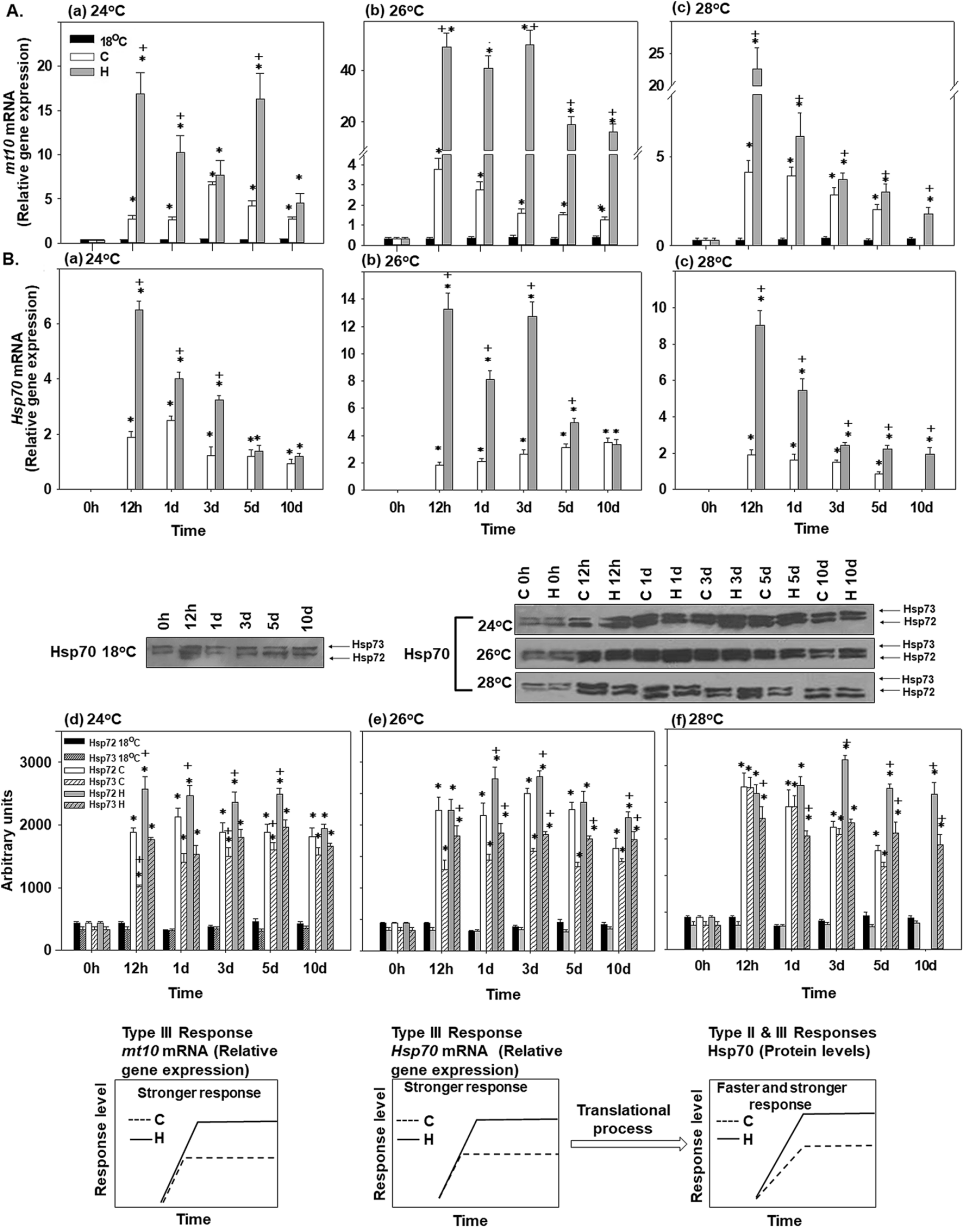
Changes in (**A)** the relative *metallothionein-10* mRNA levels [Type III (stronger) response] and in (**B**) the relative Hsp70 mRNA levels [Type III (stronger) response], and Hsp72 and 73 levels [Type II & III (faster and stronger) responses] in the mantle of heat hardened (H) and non-hardened (C) *Mytilus galloprovincialis* when exposed to 24 °C (a,d), 26 °C (b,e) and 28 °C (c,f) compared to control at 18 °C. Representative blots are shown. Blots were quantified using scanning densitometry. Full-length blots/gels are presented in Supplementary Fig. 2. Values are means ± SD, n = 8 preparations from different animals. *p < 0.05 compared to day 0, ^+^*p* < 0.05 compared to non-hardened (C) mussels.

Transcript levels of *mt-10* increased above the control (18 °C) baseline at all experimental temperatures in the hardened and non-hardened mussels (Fig. 6Aa–c). The amplitude of this increase was consistently higher in the hardened mussels compared with their non-hardened counterparts at all experimental time points. At 28 °C, *mt-10* expression levels decreased gradually during the prolonged exposures; this tendency was not observed at 24 °C or 26 °C (Fig. 6Aa–c).

Statistically significant differences were observed between hardened and non-hardened specimens. Main effects of treatment and exposure time, as well as factor interactions, were significant (*p* < 0.0001).

### *Hsp70* mRNA and protein expression

*Hsp70* mRNA was upregulated to above control (18 °C) baseline levels at all experimental temperatures in the hardened and non-hardened mussels (Fig. 6Ba, b, c). The amplitude of this increase was higher in the hardened mussels than in the non-hardened mussels during the earlier stages of response to heat stress at 24 °C (days 1–3) and 26 °C (days 1–5) (Fig. 6Ba,b) and at all experimental time points at 28 °C (Fig. 6Bc). There was a decrease in *Hsp70* mRNA during the later stage of experimental exposures (5–10 days at 24 °C, 10 days at 26 °C and 3–10 days at 28 °C) in the hardened mussels but not in their non-hardened counterparts (Fig. 6Ba–c).

Protein levels of the two detected Hsp70 isoforms (Hsp73 and Hsp72) increased during the first 12 h of experimental exposures and remained elevated throughout 10 days at 24, 26 and 28 °C (Fig. 6Bd–f). This pattern was found in both hardened (H) and non-hardened (C) mussels. In general, the hardened (H) mussels showed higher levels of Hsp73 and Hsp72 compared to non-hardened (C) mussels at all exposure temperatures.

Statistically significant differences were observed between hardened and non-hardened specimens. Main effects of treatment and exposure time, as well as factor interactions, were significant (*p* < 0.0001).

## Discussion

### Bioenergetics

Our present work demonstrates enhanced thermotolerance in heat hardened *M. galloprovincialis* as shown by their improved survival during heat exposure (particularly at the lethal temperature of 28 °C) compared to the non-hardened mussels. The enhanced thermotolerance of heat hardened mussels could in part be attributed to improved mitochondrial respiration as indicated by increased ETS activity, and elevated levels of the transcripts encoding mitochondrial ETS subunits *ND2* and *COX1* at the higher tested temperatures (26 °C and 28 °C). Notably, the dynamics of the heat-induced changes in the transcript levels and the ETS activity appear synchronized in the hardened mussels. Thus, the ETS activity, as well as *ND2* and *COX1* expression, peaked at day 5 of exposure at 26 °C in the hardened mussels. At 28 °C, an initial increase in ETS activity at 12–24 h was followed by a decline by day 3, and the second peak at day 5 was mirrored in the *COX1* transcript levels in the hardened mussels. In the non-hardened mussels, *ND2* and *COX1* mRNA levels, and ETS activity remained considerably lower than in the hardened mussels at 26 °C and 28 °C, and the dynamics of the enzymatic activity and transcriptional response was de-synchronized. Enhanced mitochondrial activity in the hardened mussels might help with ATP provision to cover high energy demand due to the rate-enhancing (Q_10_) effects of warming on the cellular ATP consumers, thereby mitigating the heat-induced energy stress and supporting energy homeostasis^9^. Other changes to mitochondrial characteristics remain to be investigated.

Despite a strong upregulation of *ND2* and *COX1* mRNA expression at 24 °C in hardened, as well as non-hardened mussels, the ETS activity decreased below baseline (18 °C) levels in both studied groups at this temperature. This implies predominant involvement of post-transcriptional mechanisms in regulation of ETS activity at 24 °C such as post-translational modifications of mitochondrial proteins^37^. Alternatively, the observed decrease in ETS activity at 24 °C might reflect damage to the mitochondria due to the elevated temperature. This explanation appears less likely because such decrease is not observed at higher temperatures (26 °C and 28 °C), at least not in the hardened mussels. Overall, moderate heat stress (24 °C) has a weak impact on the ETS activity in both experimental groups with no apparent beneficial effects of heat hardening. In contrast, warm acclimation may occur, removing excess mitochondrial capacity, as observed earlier across many ectotherms^38^. Furthermore, exposure to 24 °C does not induce elevated mortality in the hardened or non-hardened mussels. These findings indicate that 24 °C falls within the pejus (rather than the pessimum) temperature range for the studied population of *M. galloprovincialis*^3,8,9^.

On the other hand, an increase in ETS activity beyond the control levels of non-hardened mussels after the third day of exposure at sublethal temperatures is strong evidence for enhanced oxygen delivery, resulting in elevated aerobic capacity and ATP synthesis, indicating an upward shift of thermal limits and accordingly, oxygen supply during heat acclimation. The importance of ATP homeostasis in marine bivalves for defense against thermal stress has been extensively discussed by Sokolova^8,9^. Conversely, it has been pointed out that anaerobiosis is linked with lower thermotolerance capacity, which is correlated to internal hypoxia within heat stressed animals, i.e. OCLTT hypothesis^4,6^. Accordingly, thermally induced mortality in *Mytilus edulis* (Linnaeus, 1758) was related to a sharp increase in mantle succinate indicating insufficient oxygen reaching mitochondria^39^. Dunphy et al.^40^ reported that succinic acid levels were significantly higher in naïve compared to heat-hardened mussels, indicating mitochondrial perturbations. We did not determine the ATP levels in the present work, while the patterns of intermediate metabolism in hardened *M. galloprovincialis* are under investigation. However, the phosphorylation of AMPK, and hence activation, indicates lower heat sensitivity in hardened compared to non-hardened mussels. AMPK is considered a sensor of cellular energy status and its phosphorylation is stimulated by several factors including hypoxia which causes elevation in AMP/ATP ratio, indicating disturbance in ATP homeostasis^41^. The absence of AMPK phosphorylation in the hardened mussels when exposed to sublethal temperatures is additional strong evidence for the maintenance of energetic potential and probably enhanced aerobic capacity supporting heat hardening.

From integrating the increase in ETS activity and the shifted level and timing of antioxidant stress responses, we also suggest that such an adaptive response benefits mussel thermotolerance and acclimation to heat. In accordance with the above adaptive cellular responses, *Hsp70* mRNA expression and Hsp73 and Hsp72 levels are significantly increased during hardening near sub-lethal temperatures, thus further contributing to the protein stabilization needed for increased thermal tolerance. The induced Hsp72 isoform may act as a regulator against subsequent thermal stress and supports thermal protection^42^. These results are in line with the sharp increase in the relative *Hsp70* and *Hsp90* mRNA expression levels of mussels exposed to 22 °C, indicating their ability to sense thermal stress, even before the respective cellular processes are initiated^36^. In both groups of mussels, an increased capacity of ATP synthesis at 26 °C is prerequisite for the observed elevation of Hsp synthesis as a highly energy demanded process. Furthermore, according to the OCLTT hypothesis, the HSR is most commonly observed at the organism’s critical temperatures (where survival rates are still high)^4,6^ and at which the released Hsp70, binds to the increased denatured and erroneously ordered proteins, while HSF is free to activate the *Hsp* genes^43,44^.

### Oxidative stress

Mitochondrial thermal stress commonly suppresses OXPHOS and ETS activity and elevates electron leak resulting in ROS production. These changes can result in energy deficiency due to the mismatch between cellular ATP demand and mitochondrial ATP generation, and lead to oxidative injury if the antioxidant system cannot cope with the increased ROS production^7,13^. The existing protective mechanisms sufficiently deal with moderate stress (24 °C). Even though hardening increases the expression and activity of some antioxidants and Hsps at 24 °C, this does not appear to provide benefits in terms of aerobic capacity and organismal survival but may reflect thermal acclimation. In molluscs, including *M. galloprovincialis*, valve closure can serve as a behavioral mechanism that regulates the metabolic response by suppressing both aerobic respiration (and ROS production) and energy expenditure under unfavorable conditions^35,45,46^. Thus, thermally stressed *M. galloprovincialis* mussels keep their valves closed for a longer period, resulting in reduced oxygen consumption, hypometabolism, and activation of anaerobic metabolism^35,46^. In a recent investigation, we have discussed the relationship between ROS production and metabolic patterns in *M. galloprovincialis* in a hypometabolic state when exposed to 24 °C, 26 °C and 28 °C^36^. In line with other studies^47–50^, the correlation between succinate accumulation, changes in complex II in the respiratory chain, and in ROS production in mitochondria has been underlined. It has been proposed that this complex pattern switches the catalytic activity from succinate dehydrogenase to fumarate reductase at diminished oxygen levels. This transition is also the main step towards succinate accumulation in mussels under hypoxia/anoxia^51^. Importantly, Gracey and Connor^52^ showed that valve closure caused bradycardia in *M. californianus*, accompanied by accumulation of succinate, induction of several transcription factors, broadly classified as early genes (activated and transcribed within minutes of various stimuli), and isoforms of carbonic anhydrase, consistent with the association between anaerobic metabolism and tissue acidosis. During prolonged thermally induced hypoxic exposure, however, anaerobic degradation of glycogen to alternative anaerobic end products, such as acetate and propionate, could be considered another major adaptive mechanism that contributes to longer survival during sustained environmental hypoxia^53–57^.

In mitochondria, ROS are produced by Complex I and the NADH dehydrogenase component of the mitochondrial matrix side, and are detoxified by the antioxidant defense in the matrix^35^. The hypometabolic state (e.g. induced by valve closure) is characterized by reduced Krebs cycle rate and limits the supply of NADH to mitochondrial Complex I. The limited oxidation of NADH via Complex I may suppress the electron transfer through the ETS under thermal stress and contribute to the metabolic depression and low ROS production in the hardened and non-hardened mussels. Tomanek and Zuzow^58^ have shown that metabolic patterns during thermal stress are involved in the putative switch from NADH- to NADPH producing pathways in mussels. Changes in NADP^+^-dependent mitochondrial IDH and pentose–phosphate pathway suggest that there is a NADPH up-regulation in response to heat stress, while other changes in the Krebs cycle and the ETS suggest that the production of NADH is decreasing at the highest temperature. In line with the above hypothesis, Ramnanan and Storey^59^ reported that estivation-induced phosphorylation by G6PDH may enhance NADPH production for use in antioxidant defense. Also, Lama et al.^60^ showed that G6PDH is enhanced during anoxia in *Littorina littorea* (Linnaeus, 1758), possibly in an effort to produce NADPH reducing equivalents for use in the antioxidant defense.

The voluntary switch to anaerobiosis in bivalves may serve as a mechanism which reduces ROS formation, thus resulting in longer survival^8,45,52^. The latter in conjunction with the early gene expression of antioxidant enzymes^36^ may contribute to the dynamic equilibrium between ROS production and detoxification. The expression of both *CuSOD* and *MnSOD* genes indicates that both forms are involved in the mitochondrial reduction of superoxide (O^2.−^) to hydrogen peroxide (H_2_O_2_), which is then detoxified by CAT, while GR detoxifies endogenous compounds, including peroxidized lipids. Under hypoxic conditions, expression of *GST* mRNA is increased in *M. galloprovincialis* due to oxidative stress^61^. The mechanisms modulating gene expression for the antioxidant enzymes in molluscs as a response to ROS accumulation during temperature-induced hypometabolism, seem to involve several transcription factors targeted by members of MAPKs including ERKs, JNKs, and p38 MAPK^14,49^. Nevertheless, the physiological role of *ND2 and COX1* genes expression remains unclear after the third day of non-hardened mussel exposure to 24 °C. Since such a response is not followed by a concomitant increase in ETS activity, we could suggest that this response is a component of the "preparatory strategy" enabling mussels to strengthen their defense against higher intensities of subsequent thermal episodes^62^.

The above mentioned molecular and metabolic processes are reversed when mussels are exposed at temperatures beyond 26 °C which causes a more active metabolic response. Specifically, relative mRNA levels did not differ between non-hardened and hardened mussels during the first day of exposure to 26 °C, while further exposure, caused stronger gene expression in the hardened mussels compared to non-hardened ones, maintaining high mRNA levels by the 10th day. A similar pattern was observed when mussels were exposed to 28 °C. The above data clearly indicate a phenotypic change in the transcription in the *ND2* and *COX1* genes, which probably enhances the potential for mitochondria respiration compared to control (18 °C) and non-hardened mussels especially when exposed to 26 °C. In line with the above gene responses is the longer thermal acclimation and delayed mortality of mussels. However, such a molecular response cannot by itself explain longer survival after heat-shock and hardening, unless it is related to an enhanced oxidative defense or decreased proton leak and hence reduced ROS production. Furthermore, we should point out that the beneficial effects of hardening seem to take place after the third day of exposure to sublethal temperatures, a fact coinciding well with the deceleration in the mortality rate of mussels. Consequently, the question raised is which cellular mechanisms are involved in the increase of thermal tolerance in the hardened mussels when exposed to subthelal temperatures beyond 26 °C. First, the more efficient (faster and higher) antioxidant defense against increased ROS production in heat hardened compared to non-hardened mussels could be partly the answer to the above question. In both cases (non-hardened and hardened mussels), all genes for antioxidant enzymes indicate a strong response. However, the enzymatic activities seem to exhibit different kinds of responses. Specifically, SOD total enzymatic activity exhibited a faster but not stronger response. On the contrary, CAT and GR exhibited faster and stronger responses, indicating a higher capacity in hardened mussels for scavenging redundant ROS compared to non-hardened. The latter may be of crucial importance in oxidative defense which is enhanced during hardening thus enabling mussels to develop higher thermotolerance and lower mortality. In line with the *GST* and *CAT* mRNA expression, the stronger *mt-10* gene transcription in hardened *M. galloprovincialis* further supports the increased defense and tolerance of heat hardened mussels against thermal stress. Giannetto et al.^11^ reported a cytoprotective role in the physiological oxidative stress response of mt-10 and mt-20 in *M. galloprovincialis.* The family of *mt* genes demonstrates enhanced expression in many organisms after heat shock treatment^63–65^, suggesting a relationship between HSR and *mt* gene expression^66^.

### Acquired stress memory

Overall, the acquired thermal stress resistance or as it is sometimes called "acquired stress memory", is a phenomenon in which cells exposed to a mild dose of one stress can subsequently survive an otherwise lethal dose of the same or a second stress. There are several reports on the beneficial effects of this adaptive response on the thermal tolerance of several mollusc species, including the Asian green mussel *Perna viridis* (Linnaeus, 1758)^67^, blue mussel *M. edulis*^68^ and New Zealand green-lipped mussel *Perna canaliculus* (Gmelin, 1791)^40^. Moreover, warm preconditioning seems to protect against acute heat-induced respiratory dysfunction and delays bleaching in a symbiotic sea anemone^69^. Also, Wesener and Tietjen^70^ showed that independently of stress type and priming costs, the stronger primed response is most beneficial for longer stress phases of several microbial species, while the faster and earlier responses increase population performance and survival probability under short stresses. On the other hand, Pereira et al.^71^ reported that early exposure of oyster species to heat shock had little effect on the amelioration of increased temperature’s negative effects, although the survival of heat-shocked oysters was greater than non-heat shocked ones. The cellular mechanisms involved in acquired stress resistance are not known. Krebs and Loeschcke^72^ reported for *Drosophila melanogaster* (Meigen, 1830) that the acclimation to heat stress may be owed in part to the presence of large numbers of mRNA transcripts for heat-shock proteins or to the presence of the specific stress proteins. In yeast, this acquired stress resistance depends on protein synthesis during mild-stress treatment and requires the “general-stress” transcription factors that regulate induction of many environmental stress response genes^73^. In line with the above assumption, Horowitz^30,31^ reported that cells respond to extra- and intra- cellular signals by changing gene expression patterns which seem to play important roles in acclimation and that the long-term physiological responses to acclimation are determined by the molecular programs that induce the acclimated transcriptome. However, some investigations focused on the importance of the initial temperature stress which dictates the recovery time (through activation of CSR), along with the duration of the changes (i.e. a highly activated CSR may have led to more permanent changes within the cells that led to a longer-lasting heat tolerance)^62,74^. Also, the number of heat-shock bouts that an organism experiences at sublethal temperatures may determine largely how long this organism can tolerate extreme temperatures^74,75^. In this prism, Connor and Gracey^75^ reported that *M. californianus* exhibits phenotypic plasticity with respect to transcriptomic expression during cycles of aerial exposure and that it may produce a type of “heat hardening”, thus promoting homeostasis in the intertidal environment and enhanced tolerance to low-tide heating events. Moyen et al.^34^ recently reported that this adaptive strategy via phenotypic plasticity will likely prove beneficial for *M. californianus* and other mussel species under the extreme heat events projected with progressing climate change.

## Materials and methods

### Animals

The mussels *M. galloprovincialis* with a total mass of 25.82 ± 4.62 g (mean ± SD), shell length 6.42 ± 0.47 cm and shell width 3.2 ± 0.15 cm, were collected from a mussel farm located in Thermaikos Gulf, Greece (Dramouslis LTD) in late April, when the ambient sea water temperature was approximately 18 °C. Mussels were transferred to the Laboratory of Animal Physiology, Department of Zoology, School of Biology of the Aristotle University of Thessaloniki and kept in 1000 l tanks with recirculating aerated natural seawater for 1 week. Water temperature was controlled at 18 °C ± 0.5, while salinity was kept at 34‰ ± 2.85 and pH at 8.12 ± 0.05, respectively. Mussels were kept under 14/10 h light–dark photoperiod, in order to mimic the field conditions when the mussels were collected. Mussels were fed daily with 0.5% dry weight cultured microalgae *Tisochrysis lutea* (CCAP 927/14)/gr total weight of mussels. 60% of water was replaced every 2 days with filtered seawater.

#### Experimental procedures

The experimental exposures were conducted in two phases (heat hardening and acclimation phases) as described below. During hardening and acclimation phases, mussels were not fed.

##### Heat-hardening phase

**Figure 7:**
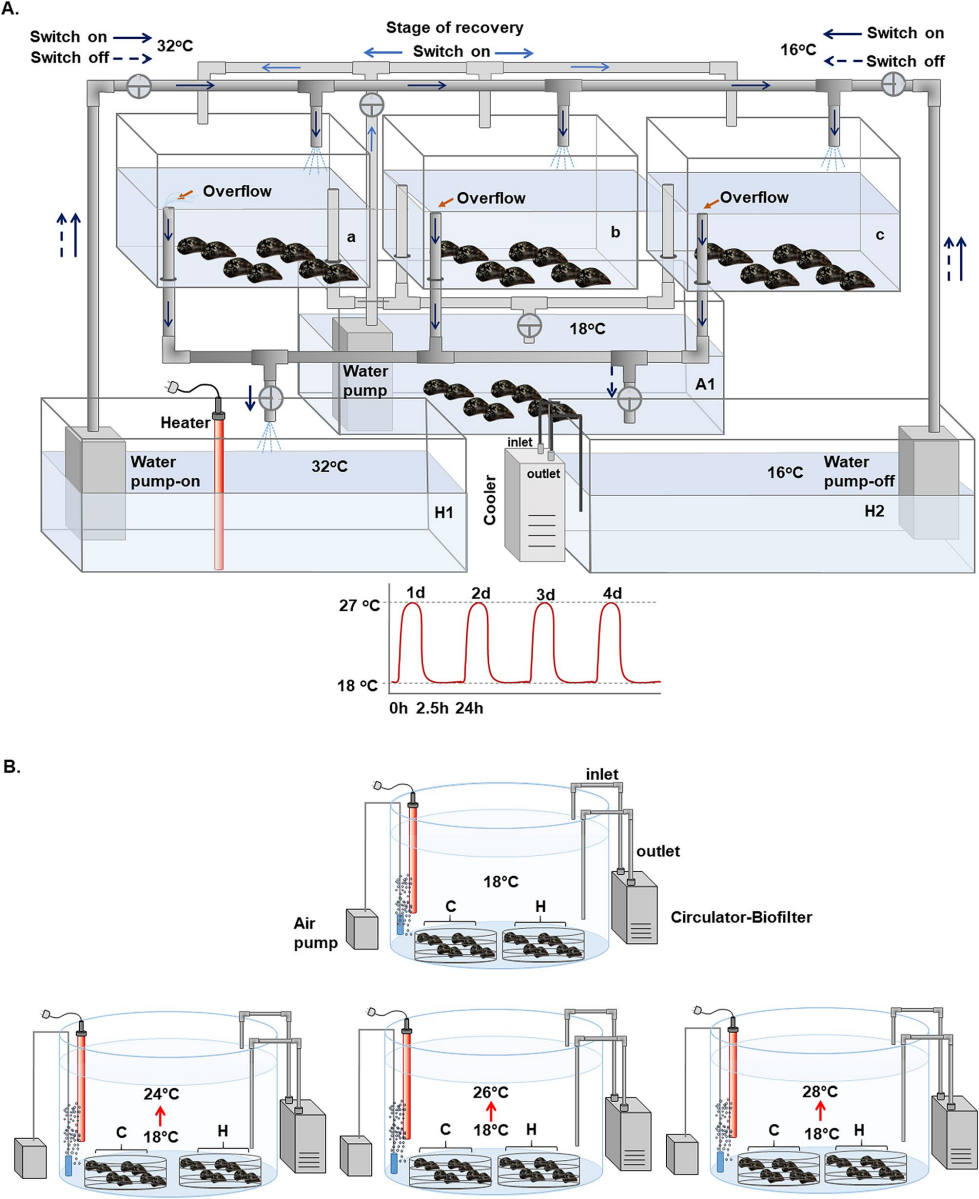
(**A**) Mussels’ hardening phase: tanks with fully aerated water at 18 °C were connected with switches with two other tanks, one with increased (32 °C) and the other with decreased (16 °C) water temperature in order to regulate the water temperature to increased (27 °C) or decreased (18 °C—control) temperatures. (**B**) Mussels’ acclimation phase: transfer of all (hardened and non-hardened) individuals in tanks with recirculating aerated natural seawater at 18 °C and consequent increase at 24 °C, 26 °C and 28 °C.

Experimental design for heat hardening exposures was based on Hutchison's ^76^ "Repeated—CTM" method, with minor modifications (Fig. 7A). Specifically, ~ 300 randomly selected mussels were divided and conditioned in three aquaria each containing 100 l aerated sea water at 18 °C (a, b and c). The sea water was recirculated via pipes connected to a 500 l tank (A1) maintained at 18 °C. Mussels were kept at 18 °C for 1 week. To determine whether a single sublethal heat-stress bout would confer improved heat tolerance during a subsequent more extreme (potentially lethal) heat-stress exposure, mussels were given a sublethal heat-stress bout of 2.5 h at 27 °C. We have shown in previous works that mortality of *M. galloprovincialis* increases significantly after exposure to 27 °C^35,36^. The studied populations of mussels currently experience similarly high temperatures (27–28 °C) in the field during summer. It has been reported that during the last decades, there has been a continuous temperature increase in the Mediterranean Sea, which is expected to rise further in the near future^77,78^. These climate projections for Mediterranean and Aegean seas include also an increased intensity in the frequency of the heat waves with temperatures exceeding 27 °C. Consequently, the experimental design aimed to simulate the projected changes in sea water temperature in the area under study. To increase the sea water temperature in exposure tanks (a, b, and c on Fig. 7A), water flow was switched to a heater tank containing sea water at 32 °C (H1). As reported elsewhere, this treatment permits organismal temperature to rise without time lag^79^. Once temperature reached 27 °C, the water flow from tank H1 was stopped and the mussels were exposed to 27 °C for 2.5 h. Thereafter, the water flow was switched to tank H2 containing sea water at 16 °C that permitted a quick drop of the water temperature in exposure tanks (a, b and c) to 18 °C. Afterwards, water flow stopped, and mussels were left to recover at 18 °C for 24 h. This heat-stress bout (including the thermal shock and recovery phase) was repeated four times. The mussels exposed to this treatment are later called heat-hardened mussels. Control mussels were maintained in three aquaria (each containing 100 l aerated sea water) at 18 °C during this time.

##### Acclimation phase

After the completion of heat-hardening treatments, both control (non-hardened) mussels (group C) and hardened mussels (group H) were transferred to four 500 l tanks (50–60 individuals from each group per tank) with recirculating aerated natural seawater at 18 °C and left to recover for four days. Group C and group H mussels were placed respectively in different baskets within each tank. Thereafter, water temperature of the three tanks was increased (1 °C/h) to 24, 26 and 28 °C, respectively. As reported elsewhere, this treatment permits organismal temperature to simultaneously rise with the test temperature without time lag^79^. Mussels maintained in the fourth tank were kept at 18 °C and used as controls (Fig. 7B). All different tanks were run in triplicates.

#### Tissue sampling and water quality monitoring

Individuals (n = 8 at each time point) from hardened and non-hardened groups were collected from each tank at 12 h, 1, 3, 5 and 10 days after the target temperature (24, 26 or 28 °C) was reached. As we have reported elsewhere^36^, mantle exhibited higher aerobic capacity and more intense physiological stress response compared to PAM. Therefore, the mantle was removed, immediately frozen in liquid nitrogen and stored at − 80 °C for later analysis.

Physicochemical water parameters (Table 1) were measured daily as follows: salinity (g l^−1^), O_2_ (mg l^−1^) and pH by using Consort C535, Multiparameter Analysis Systems (Consort, bvba, Turnhout, Belgium) while NH_3_ (μg l^−1^), NO_2_^−^ (μg l^−1^) and NO_3_^−^ (μg l^−1^) were analyzed by using commercial kits by Tetra (Tetra Werke, Melle, Germany).

**Table 1:** Mean values of sea water parameters.

	Temperature (^o^C)	Salinity (g l^−1^)	pH	O_2_ (mg l^−1^)	NH_3_ (mg l^−1^)	NO_2_^−^ (mg l^−1^)	NO_3_^−^ (μg l^−1^)
18 °C	18 ± 0.1	33.2 ± 0.03	8.04 ± 0.02	7.9 ± 0.06	0.2 ± 0.01	0.2 ± 0.01	< 12.5
24 °C	24 ± 0.3	33.4 ± 0.03	8.03 ± 0.02	7.8 ± 0.07	0.2 ± 0.01	0.3 ± 0.02	14 ± 0.6
26 °C	26 ± 0.4	33.3 ± 0.01	8.02 ± 0.01	7.8 ± 0.05	0.2 ± 0.02	0.3 ± 0.01	14 ± 0.3
28 °C	28 ± 0.3	33.2 ± 0.04	8.02 ± 0.01	7.8 ± 0.07	0.3 ± 0.02	0.2 ± 0.02	13 ± 0.5

Sea water parameters as measured in all experimental conditions. Values are presented as means ± SD (n = 10).

### Analytical procedures

#### SDS/PAGE and immunoblot analysis

The preparation of tissue samples for SDS-PAGE and the immunoblot analysis are based on well-established protocols (e.g.^35^). In the present study, equivalent amounts of proteins (50 μg per sample) were separated on 10% and 0.275% (w/v) acrylamide and bisacrylamide slab gels and transferred electrophoretically onto nitrocellulose membranes (0.45 μm, Schleicher and Schuell, Keene N. H. 03431, New Hampshire USA). The nitrocellulose membranes were stained with Ponceau dye to assure good transfer quality and equal protein loading. Antibodies used were monoclonal anti-phospho AMPK (2535, Cell Signaling, MA Beverly, USA) and monoclonal Anti-Heat Shock Protein 70 (H5147, Sigma, Darmstadt, Germany).

#### Determination of activities of antioxidant enzymes in the tissue homogenates

Crude extracts for determination of the activities of antioxidant enzymes were obtained as described in Salach^80^. Briefly, the frozen mantle tissues were homogenized in ice-cold phosphate buffer (50 mM), adjusted to pH 7.4, using an Omni international homogenizer (Thane, USA) at 22,000 rpm for 20 s. The homogenates were centrifuged at 2000*g* at 4 °C for 15 min. The supernatants were freeze-thawed thrice to fully disrupt the organelle membranes and centrifuged at 6000*g* at 4 °C for 15 min. The resulting supernatants were used for assaying the activities of antioxidant enzymes. SOD (total activity of mitochondrial Mn- and cytosolic Cu/Zn-SOD, SOD EC 1.15.1.1) was assayed by monitoring the inhibition of NADH oxidation using β-mercaptoethanol in the presence of EDTA and Mn as a substrateas described in Paoletti and Mocali^81^. The changes in the absorbance of NADH at 340 nm per min were determined (ε = 6.22 mM^−1^ cm^−1^). CAT *V*_*max*_ (CAT, EC 1.11.1.6) was determined following the changes in the absorbance of H_2_O_2_ at 240 nm (extinction coefficient ε = 0.0394 mM^−1^ cm^−1^) according to Cohen et al.^82^. GR *V*_*max*_ (GR, EC 1.8.1.7) was determined by a NADPH-coupled assay according to Carlberg and Mannervik^83^, following the changes in the absorbance of NADPH (ε = 6.22 mM^−1^ cm^−1^). All enzymatic activities were determined at 18 °C using a Hitachi 150–20 recording spectrophotometer with water-jacketed cell. The *V*_*max*_ of the enzymes was expressed as units per gram of wet tissue mass.

#### Determination of ETS activity

ETS activity was determined according to Haider et al.^84^. Briefly, 10 mg powdered tissue was added to 500 µl homogenizing buffer (Tris–HCl 0.1 M, poly vinyl pyrrolidone 0.15% (w/v), MgSO_4_ 153 µM, Triton X-100 0.2% (w/v); adjusted to pH 8.5). After centrifugation (3000*g*, 4 °C, 10 min), 28.5 µL from each extract was added to 85.5 µL buffered substrate solution (Tris–HCl 0.13 M and Triton X-100 0.3% (w/v); adjusted to pH 8.5) and 29 µL NAD(P)H solution (NADH 1.7 mM and NADPH 250 µM). For blank readings, NAD(P)H solution was replaced with 27 µL of 5 M KCN and 2 µL of 1 mM rotenone. The reaction was initiated by the addition of 57 µL of 8 mM iodonitrotetrazolium (INT). Absorbance was measured at 18 °C for 10 min at 490 nm. The ETS activity was calculated from the rate of the formazan formation using ɛ_490 nm_ = 15,900 M^−1^ cm^−1^ and a stoichiometry of 1 µmol of O_2_ to 2 µmol of formazan.

#### Determination of mRNA expression

For estimation of mRNA expression profiles, a quantitative real-time PCR was performed. Total RNA was isolated from homogenized mantle tissue using Tri-Reagent (Sigma, St. Louis, MO, USA) following the manufacturer’s instructions. Approximately 100–150 ng RNA was utilized for first strand cDNA synthesis using the PrimeScript kit (Takara, Gunma, Japan) and the oligodT primers. With the use of the Sensi-FAST SYBR No-ROX mixture (Bioline, London, UK) in a Bio-Rad CFX96 real-time PCR thermocycler, the mRNA levels of the target genes were determined including *COX1*, NADH dehydrogenase subunit 2 (*ND-2*), *hsp70*, *mt-10*, *GST*, Mn superoxide dismutase (*Mn-SOD*), Cu/Zn superoxide dismutase(*Cu/Zn-SOD*), and *CAT* (*cat*). Primers used for amplification of the aforementioned genes were as described in Feidantsis et al.^36^ except for *COX1* and *ND-2* which were described in Woo et al.^61^. The *actin* gene, used as reference, was amplified using the primers actin-F and actin-R (Accession No: AF157491). The sequences of used primers are shown in Table 2. Relative quantification was achieved by comparing the cycle threshold values (CT) of the target genes with the CT values of the *actin* using the ΔΔCT quantification algorithm.

**Table 2:** Primers used for the estimation of gene expression profiles.

Gene	Sequence	Amplicon size	Accession number	Reference
COX1 F	5′-GTGTCTTCTTATGGGTCTG-3′	211	FJ890849	Woo et al. ^61^
COX1 R	5′-GCTATAAACATGCTTTCTCC-3′			
ND2 F	5′-TGGTGTTTTCCTCTACACTC-3′	210	FJ549901	Woo et al. ^61^
ND2 R	5′-AGGGTCTTATTACCCGCACT-3′			
Actin F	5′-CGACTCTGGAGATGGTGTCA-3′	153	AF157491	Moreira et al. ^85^
Actin R	5′-GCGGTGGTTGTGAATGAGTA-3′			

### Statistics

Changes in Hsp70 levels, ETS activity, antioxidant enzymes activities and relative mRNA expressions were tested for significance at the 5% level (*p* < 0.05) by using one-way analysis of variance (ANOVA) (GraphPad Instat 3.0) for statistically significant differences between examined groups and two-way (GraphPad Prism 5.0) analysis of variance (ANOVA) for the significance of factors tested each time, with sampling days and treatment as fixed factors. Post-hoc comparisons were performed using the Bonferroni test. Values are presented as means ± S.D.

## Supplementary Information

Supplementary Information.

## Abbreviations

AMPK: AMP activated protein kinase
ATP: Adenosine triphosphate
CAT: Catalase
G6PDH: Glucose-6-phosphate dehydrogenase
CSR: Cellular stress response
CTM: Critical thermal maxima
CuSOD: Cu superoxide dismutase
COX1: Cytochrome c oxidase subunit 1
ETS: Electron transport system
EDTA: Ethylenediaminetetraacetic acid
GR: Glutathione reductase
GST: Glutathione *S*-transferase
HSF: Heat shock factor
Hsp70: Heat shock protein
HSR: Heat shock response
IDH: Isocitrate dehydrogenase
NADH: Nicotinamide adenine dinucleotide
ND2: NADH dehydrogenase subunit 2
NADPH: Nicotinamide adenine dinucleotide phosphate
mt10: Metallothionein 10
MnSOD: Mn superoxide dismutase
OXPHOS: Oxidative phosphorylation
OCLTT: Oxygen and capacity limited thermal tolerance
PAM: Posterior adductor muscle
ROS: Reactive oxygen species
SOD: Superoxide dismutase
Mn: Manganese
