# Supplementary material for: Heat hardening enhances mitochondrial potential for respiration and oxidative defence capacity in the mantle of thermally stressed Mytilus galloprovincialis
Source: Sci Rep. 2021 Aug 24;11:17098. doi: 10.1038/s41598-021-96617-9 (PMC8384858; doi:10.1038/s41598-021-96617-9)

Heat hardening enhances mitochondrial potential for respiration and oxidative defence capacity in the mantle of thermally stressed *Mytilus galloprovincialis*

Ioannis Georgoulis[1], Konstantinos Feidantsis[1], Ioannis A. Giantsis[2], Asimina Kakale[1], Christian Bock[3], Hans O. Pörtner[3], Inna M. Sokolova[4] and Basile Michaelidis[1]*

[1]Laboratory of Animal Physiology, Department of Zoology, School of Biology, Aristotle University of Thessaloniki, GR-54124 Thessaloniki, Greece

[2]Department of Animal Science, Faculty of Agricultural Sciences, University of Western Macedonia, 53100 Florina, Greece

[3]Alfred-Wegener-Institut, Helmholtz-Center for Polar and Marine Research, Integrative Ecophysiology, Postfach 120161, 27515 Bremerhaven, Germany

[4]Department of Marine Biology, Institute for Biological Sciences, University of Rostock, A.-Einstein Str., 3, 18055 Rostock, Germany

***To whom correspondence should be addressed: michaeli@bio.auth.gr**

Figure S1: The complete original immunoblots shown in Figure 2 are presented in order below. The individual parts comprising Figure 2 are specified using black boxes.

Cropped blots in main paper

phospho AMPK

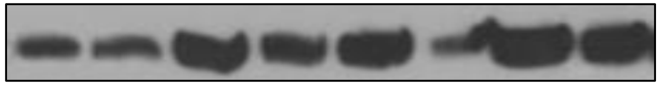

24ºC

phospho AMPK

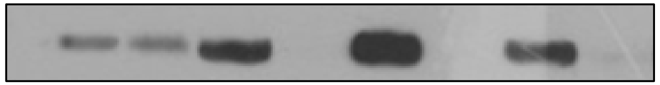

26ºC

phospho AMPK

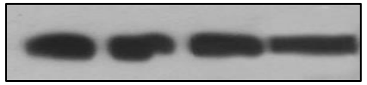

18ºC

phospho AMPK

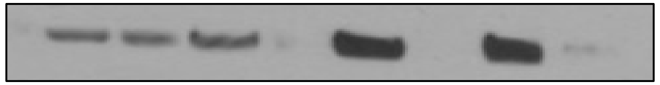

28ºC

Original blots in supplementary information

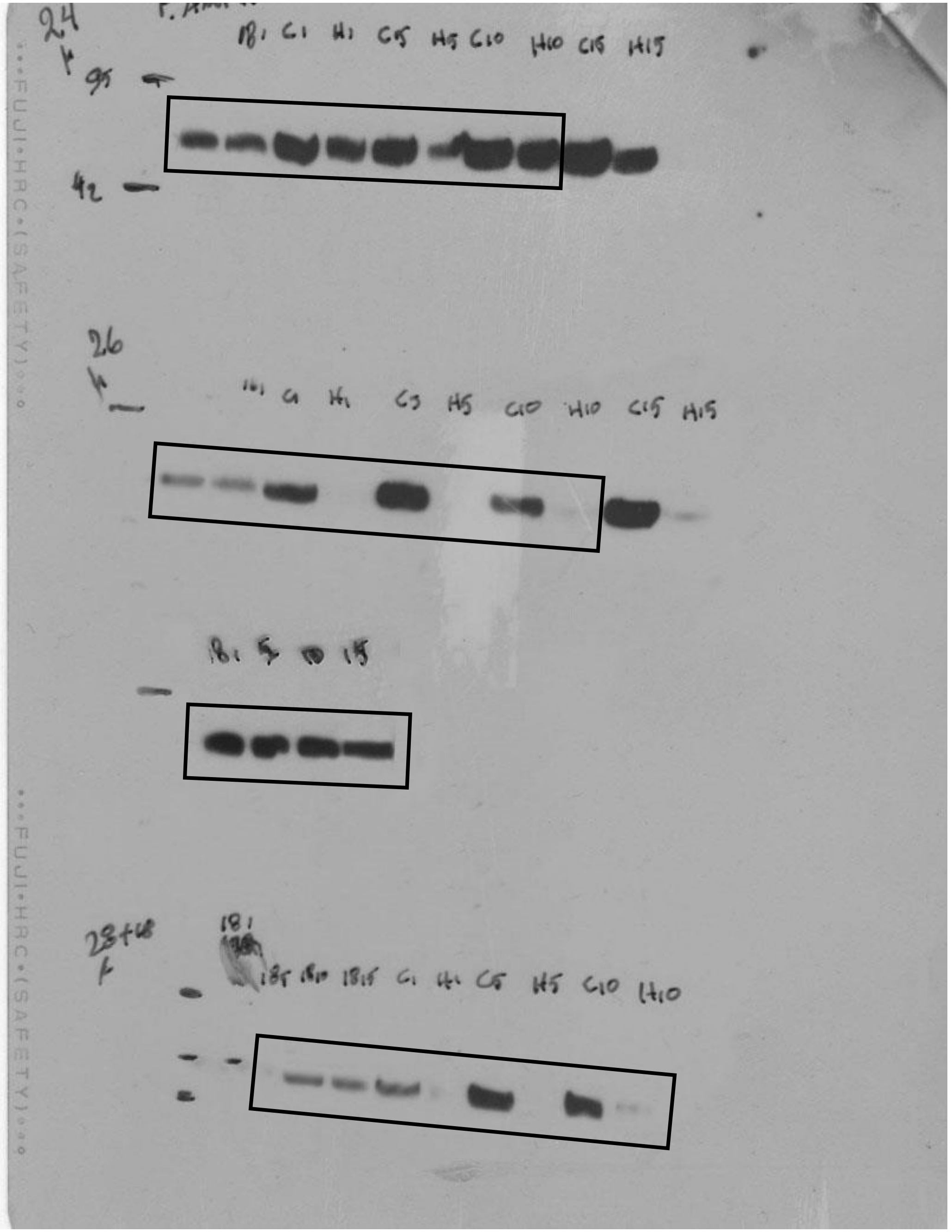

Figure S2: The complete original immunoblots shown in Figure 6 are presented in order below. The individual parts comprising Figure 6 are specified using black boxes.

Original blots in supplementary information

Cropped blots in main paper

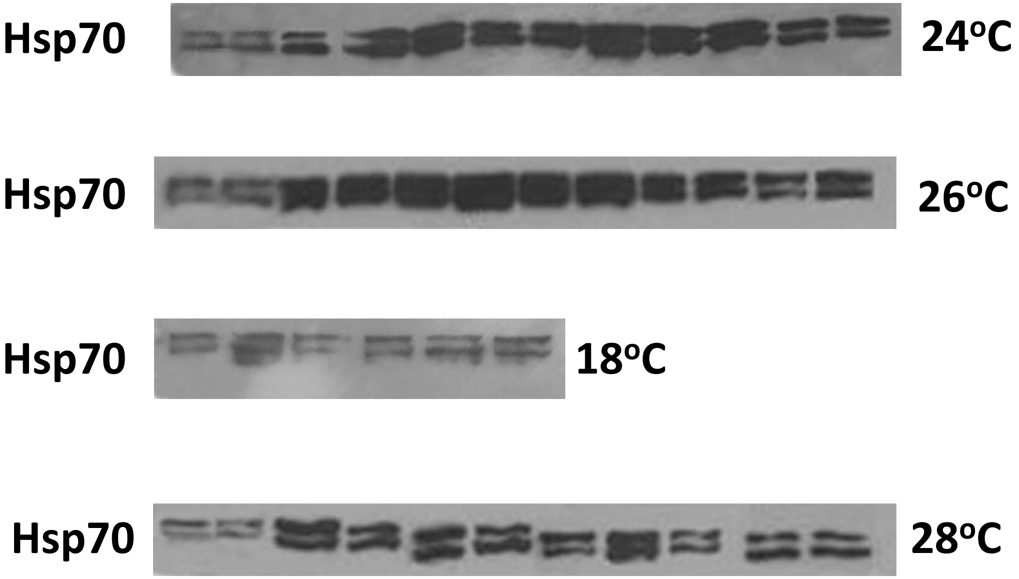

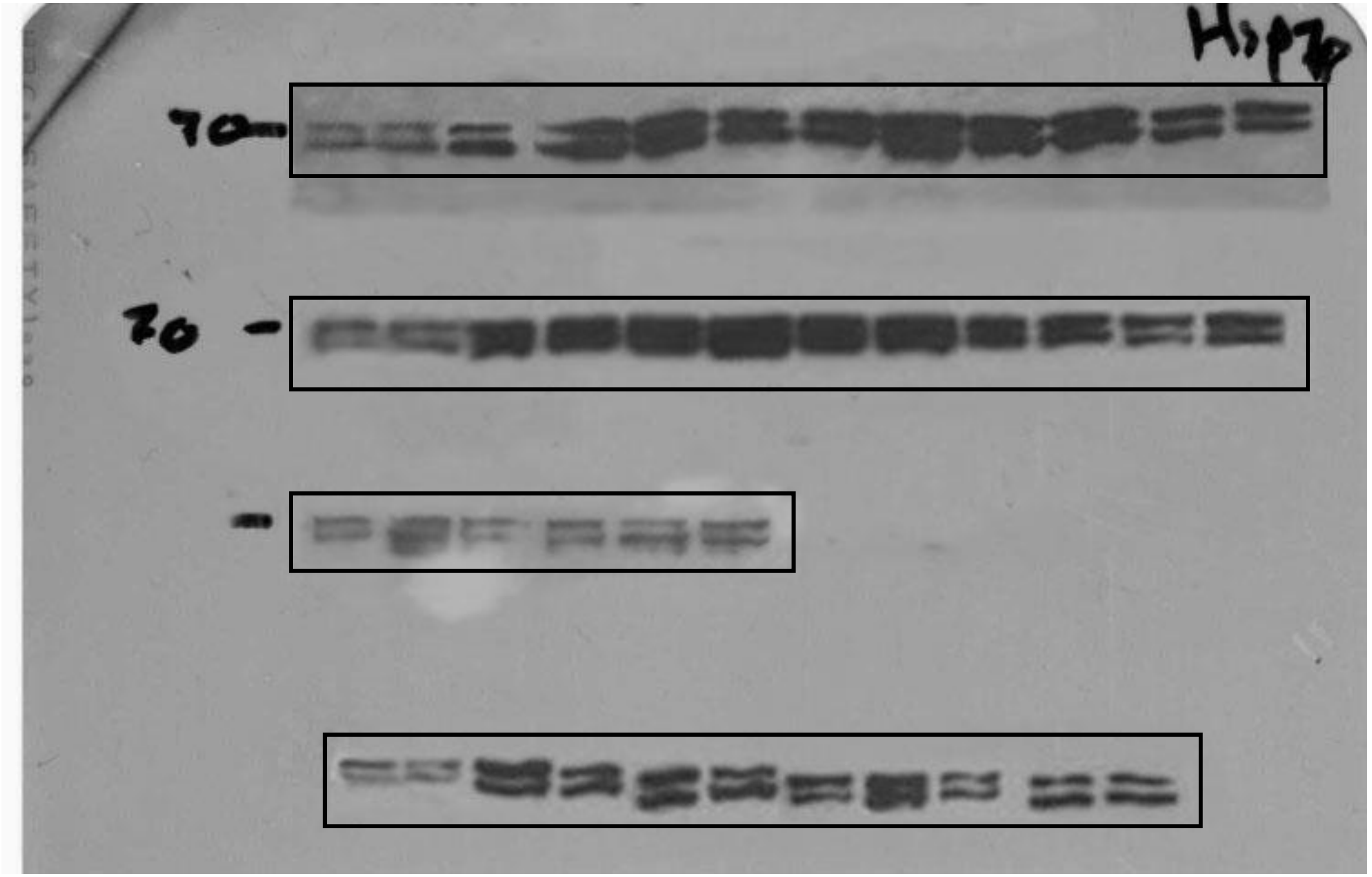

Supplement: Supplementary file 1 — Supplementary Information. [file 41598_2021_96617_MOESM1_ESM.pdf]
